# Change in Long-Spacing Collagen in Descemet's Membrane of Diabetic Goto-Kakizaki Rats and Its Suppression by Antidiabetic Agents

**DOI:** 10.1155/2008/818341

**Published:** 2008-09-02

**Authors:** Yoshihiro Akimoto, Hajime Sawada, Mica Ohara-Imaizumi, Shinya Nagamatsu, Hayato Kawakami

**Affiliations:** ^1^Department of Anatomy, Kyorin University School of Medicine, Mitaka, Tokyo 181-8611, Japan; ^2^Department of Anatomy, Yokohama City University School of Medicine, Kanazawa Ku, Fukuura 3-9, Yokohama, Kanagawa 236-0004, Japan; ^3^Department of Biochemistry, Kyorin University School of Medicine, Mitaka, Tokyo 181-8611, Japan

## Abstract

We examined changes in the ultrastructure and localization of major extracellular matrix components, including 5 types of collagen (type I, III, IV, VI, and VIII), laminin, fibronectin, and heparan sulfate proteoglycan in Descemet's membrane of the cornea of diabetic GK rats. In the cornea of diabetic GK rats, more long-spacing collagen fibrils were observed in Descemet's membrane than in the membrane of the nondiabetic Wistar rats. Both GK and Wistar rats showed an age-dependent increase in the density of the long-spacing collagen. Immunoelectron microscopy showed that type VIII collagen was localized in the internodal region of the long-spacing collagen, which was not labelled by any of the other antibodies used. The antidiabetic agents nateglinide and glibenclamide significantly suppressed the formation of the long-spacing collagen in the diabetic rats. Long-spacing collagen would thus be a useful indicator for studying diabetic changes in the cornea and the effect of antidiabetic agents.

## 1. INTRODUCTION

Corneal keratopathy is one of the diabetic complications. Clinically, the diabetic cornea often shows superficial punctate keratopathy and persistent epithelial defects and recurrent epithelial erosion that are considered to be a form of diabetic keratoepitheliopathy. Various degrees of epithelial disturbance take place in the diabetic cornea. Thickening of epithelial basement membrane in the diabetic cornea has been extensively studied [1, 2]. In a previous study, we showed that hemidesmosomes in the epithelial basal cells were decreased in number in the diabetic rats and that the basement membrane detached from the epithelial basal cells [3]. Also, the content of O-GlcNAc-modified proteins was found to be increased in the corneal epithelium as well as in the nerves, kidneys, and pancreas of diabetic rats [3–5].

The diabetic corneal endothelium has been shown by speculum-aided microscopy to have morphological abnormalities such as polymorphism [6]. The endothelial cells vary in cell shape and in cell area in the diabetic rat and human cornea [7, 8]. At the posterior side of the cornea, a thick basement membrane called Descemet's membrane is located adjacent to the endothelium. In the normal human cornea, long-spacing collagen, which is cross-striated fiber bundle, is located only in the anterior-banded zone of Descemet's membrane [9, 10]. However, in the cornea of normal Sprague-Dawley rats, there is no such collagen in Descemet's membrane [11]. Although the normal rat and human Descemet's membranes differ in this regard, in the corneas of both diabetic human patients and Streptozotocin-induced diabetic rats, unusual long-spacing collagen was observed scattered in Descemet's membrane [11, 12].

The spontaneously diabetic Goto-Kakizaki (GK) rat is a nonobese model of type 2 diabetes that was developed by the selective breeding of glucose-intolerant Wistar rats [13–15]. In the eyes of GK rats, various abnormalities have been reported, including decreased retinal microcirculation [15], elevated levels of vascular endothelial growth factor [16], nitric oxide synthase activity in the retina [17], delayed wound closure, as well as phenotypic changes in the corneal epithelium [18]. However, little attention has been paid to Descemet's membrane in GK rats. To investigate Descemet's membrane in terms of the pathogenesis of diabetes mellitus, in the present study we examined the ultrastructural morphology, and immunohistochemically determined the composition of Descemet's membrane in the cornea of diabetic GK rats in comparison with normal Wistar rats. Furthermore, we examined if the morphological change detected could be prevented by antidiabetic agents. Our findings revealed that unusual long-spacing collagen appeared and increased in content rapidly with aging in the Descemet's membrane of the diabetic rat cornea, and that its appearance could be suppressed by the antidiabetic agents.

## 2. MATERIALS AND METHODS

### 2.1. Animals and tissues

All experimental procedures using laboratory animals were approved by the Animal Care and Use Committee of Kyorin University School of Medicine. The corneas of 15-, 33-, and 62-week-old male (*n* = 6 for each age) Goto-Kakizaki rats and Wistar rats (as normal controls), obtained from Kurea (Tokyo, Japan), were used in the present study. Rats were housed under 12-hour light: 12-hour dark cycle and given free access to food and water. Serum glucose levels in Wistar and GK rats, which were measured after an overnight fast, were, respectively, 158.0 ± 12.0 and 375.9 ± 11.6 (mean ± SEM) mg/dL at 15 weeks, 118.5 ± 10.5 and 333.8 ± 22.4 mg/dL at 33 weeks, and 167.0 ± 28.6 and 314.9 ± 48.7 mg/dL at 62 weeks. As reported previously [5], serum insulin levels were also higher in the GK rats.

### 2.2. Antibodies

A monoclonal antibody (clone 9H3) against Type VIII collagen and a monoclonal antibody (clone 15B6) against Type VI collagen were prepared and characterized as described previously [19, 20]. Polyclonal antibody against laminin and type IV collagen were purchased from EY laboratory (San Meteo, Calif, USA) and LSL (Tokyo, Japan), respectively. Monoclonal antibody against heparan sulfate proteoglycan and fibronectin were purchased from Upstate Biotechnology (Lake Placid, NY, USA) and Chemicon International (Temecula, Calif, USA), respectively. Alexa568-conjugated donkey anti-rabbit or mouse IgG and SYBR-Green I were obtained from Molecular Probes (Eugene, Ore, USA).

### 2.3. Immunohistochemical localization

Immunofluorescence observation for localization of components of extracellular matrix was performed as described earlier [21]. Corneas were fixed in 4% paraformaldehyde in 0.1 M phosphate buffer (pH7.3) for 1 hour at 4°C. After having been washed with PBS, the specimens were embedded in OCT compound (Miles; Elkhart, Ill, USA). Frozen sections (4 *μ*m thick) were made, washed with PBS, and incubated for 10 minutes in 5% BSA in PBS. The sections were then incubated with anti-extra-cellular matrix component antibody for 1 hour at room temperature, washed with PBS, and subsequently incubated with Alexa 568-conjugated donkey anti-rabbit or mouse IgG antibody (1:200). Nuclei were stained with SYBR-Green I (1:500). After a final wash with PBS, the specimens were mounted in 90% glycerol-0.1 M Tris-HCl buffer (pH8.5) containing 0.5 mM p-phenylene diamine, and observed under a laser scanning confocal microscope (LSM510, Zeiss, Mass, USA). For a control experiment, the specimens were incubated with normal rabbit or mouse IgG or with 0.1% BSA-PBS alone instead of the primary antibodies. No positive staining was observed in the control experiment (data not shown).

### 2.4. Electron microscopy

Corneas were fixed in phosphate-buffered 2.5% glutaraldehyde (pH7.4). Strips of cornea were taken from the central part of the cornea, and were postfixed in 1% O_s_O_4_ in 0.1 M phosphate buffer (pH7.4), and dehydrated with graded alcohols. After immersion in propylene oxide, the specimens were embedded in Epon 812. Ultrathin sections were cut perpendicular to the epithelium, doubly stained with uranyl acetate and lead citrate, and examined with a transmission electron microscope, TEM-1010 (JEOL, Tokyo, Japan).

### 2.5. Immunoelectron microscopy

Immunoelectron microscopic observation for localization of components of extracellular matrix was performed as described earlier [22]. Fixation was carried out in the same way as for light microscopy. Ultrathin frozen-sections were cut at −90 to −100°C. The sections were washed with PBS and pretreated with 1% BSA in PBS for 10 minutes. After a PBS rinse, they were incubated with the desired antibodies for 1 hour, washed with PBS, and incubated with colloidal gold-conjugated goat anti-rabbit or mouse IgG antibodies for 1 hour. After another wash with PBS, the sections were refixed in 2% glutaraldehyde-0.1 M phosphate buffer, pH7.4, and embedded in a mixture of methylcellulose, polyethyleneglycol, and uranyl acetate.

### 2.6. Antidiabetic agents and experimental design

The administration of antidiabetic drugs was started at 8 weeks old. Nateglinide (50 mg/kg) or glibenclamide (2 mg/kg) was suspended in 0.5% methylcellulose and administered to GK rats via a stomach tube in volume of 10 mL/kg [23–25]. These doses of the antidiabetic agents were chosen from the data on their suppressive effects on the peak blood glucose levels after oral sucrose or glucose loading of fasted normal rats for 15 weeks [23, 26]. GK rats were fed twice daily (9:00 and 16:00) for 1 hour and were given nateglinide or glibenclamide orally just before each meal. Control rats were treated with 0.5% methylcellulose alone (the vehicle).

### 2.7. Statistical analysis

Results were expressed as the mean ± standard deviation, and the statistical analysis was done by the use of the unpaired Student's *t*-test. Differences were defined as significant at *P* < .05.

## 3. RESULTS

### 3.1. Changes in ultrastructural morphology of Descemet's membrane in the cornea of diabetic and normal rats with aging

**Figure 1:**
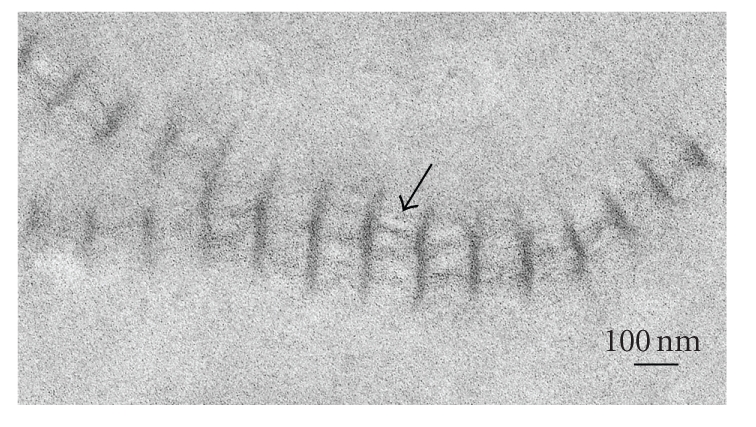
Electron microscopic image of long-spacing collagen present in Descemet's membrane of a diabetic GK rat. Arrow indicates a rod-like structure in the internodal region. Scale bar, 100 nm.

**Figure 2:**
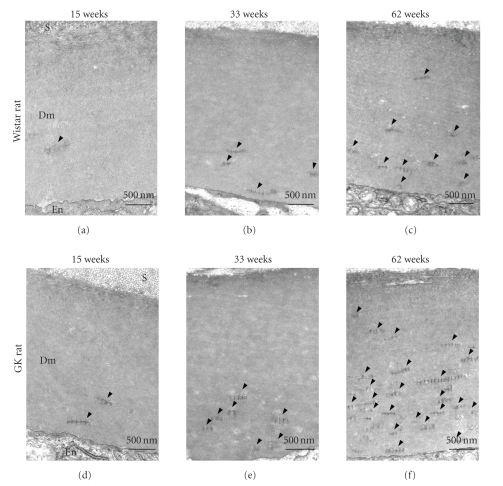
Electron micrographs of Descemet's membrane of Wistar (a), (b), (c) and GK (d), (e), (f) rat corneas at 15 (a), (d), 33 (b), (e), and 62 (c), (f) weeks. Long-spacing collagen (arrowheads) accumulates with increasing age. Dm: Descemet's membrane; En: endothelium; S: stroma. Scale bar: 500 nm.

**Figure 3:**
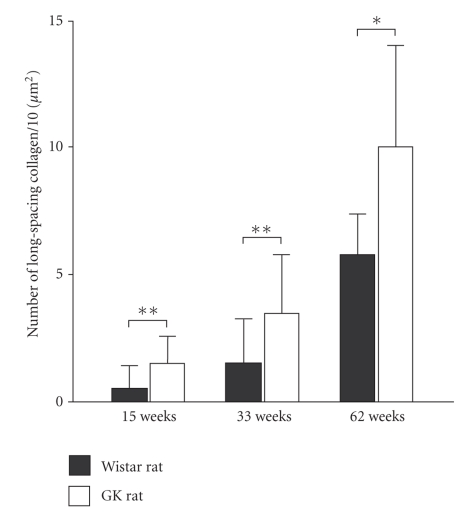
Number of long-spacing collagen fibrils per 10** *μ*m^2^ of Descemet's membrane in nondiabetic Wistar rat (▪) and diabetic GK rat (□) corneas. In the diabetic cornea, the long-spacing collagen in Descemet's increases more rapidly than in the normal cornea with aging. Data are presented as the mean ± SD (*n* = 6).*P < .05, **P < .01.

The ultrastructure of Descemet's membrane in the cornea in 15-, 33-, and 62-week-old diabetic and nondiabetic rat corneas was examined by electron microscopy. The thickness of the membrane remained unchanged in both groups. In the 15-week-old rat cornea, abnormal collagen fibril bundles (long-spacing collagen) were frequently observed in Descemet's membrane of diabetic rats, whereas they were observed less so in that of the normal rats. Figure 1 shows electron microscopic images of the long-spacing collagen. The banding pattern was wide, averaging 110–120 nm. A rod-like structure was observed in the internodal region of typical long-spacing collagen (Figure 1). The size and number of long-spacing collagen molecules increased with aging in both the normal and diabetic Descemet's membrane (Figures 2 and 3). At 62 weeks, the average length of the long-spacing collagen was 0.25–1.0 *μ*m in normal rats and 0.5–2.15 *μ*m in the diabetic ones. In the diabetic cornea, the number of long-spacing collagen fibrils in the membrane increased more than in the normal cornea with aging. The density of these collagen fibrils was higher adjacent to the endothelium and lower toward the stroma (Figure 2).

### 3.2. Immunohistochemical localization of type VIII collagen

**Figure 4:**
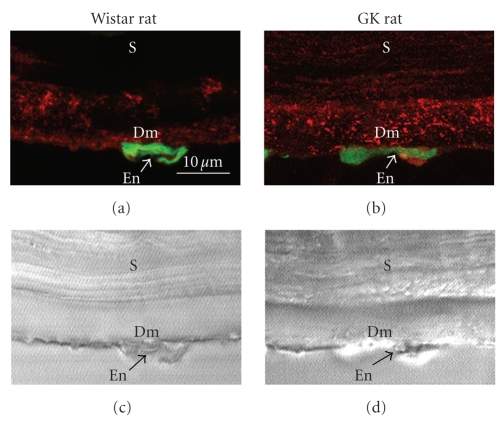
Immunofluorescence localization of type VIII collagen (red color) in Descemet's membrane of the cornea from 62-week-old rats (a), (b) and Nomarsky microscopic images of the same optical fields (c), (d). (a), (c) Wistar rat. (b), (d) GK rat. Green color (SYBR-Green I) indicates the nuclei of endothelial cells. Dm: Descemet's membrane; En: endothelial cell; S: stroma. Scale bar: 10 *μ*m.

It was earlier shown that type VIII collagen is localized in Descemet's membrane [19]. So we examined the localization of type VIII collagen in the rat cornea by laser confocal scanning microscopy (Figure 4). Whereas weak staining was observed in Descemet's membrane of the normal cornea, more intense punctate staining was observed in that of the diabetic one (Figure 4).

### 3.3. Immunoelectron microscopic localization of type VIII collagen

**Figure 5:**
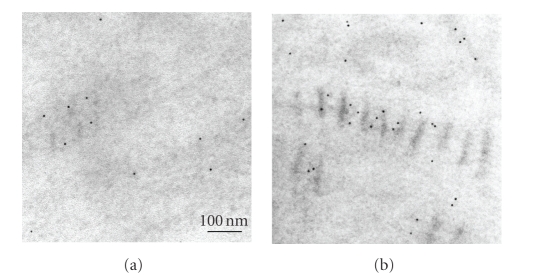
Immunoelectron-microscopic detection of type VIII collagen in Descemet's membrane by the colloidal gold labeling method. (a) In the cornea of a 15-week-old Wistar rat, the colloidal gold label is distributed diffusely in the membrane. (b) In the diabetic cornea of a 15-week-old GK rat, the gold marker is mainly observed in the internodal region of the long-spacing collagen. Scale bar, 100 nm.

Localization of type VIII collagen in Descemet's membrane was examined immunoelectron-microscopically by using the colloidal-gold labeling method (Figure 5). In the normal cornea, the colloidal gold was detected diffusely in Descemet's membrane (Figure 5(a)). In the diabetic cornea, however, it was found in the region between bands of the long-spacing collagen (Figure 5(b)). This localization of type VIII collagen in the diabetic rat is consistent with that reported previously for the diseased human Descemet's membrane [9, 10].

### 3.4. Localization of other components of extracellular matrix

**Figure 6:**
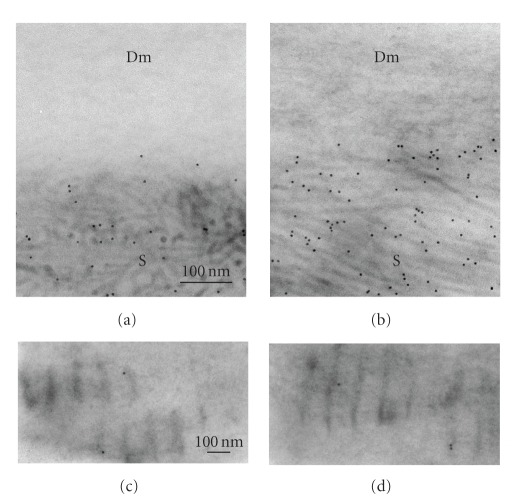
Immunoelectron-microscopic localization of type VI collagen in Descemet's membrane by the colloidal gold labeling method. In both normal and diabetic corneas, the label was detected mostly in the stroma, with little found in Descemet's membrane (a), (b) or in the long-spacing collagen (c), (d). Dm: Descemet's membrane; S: stroma. Scale bar: 100 nm.

**Figure 7:**
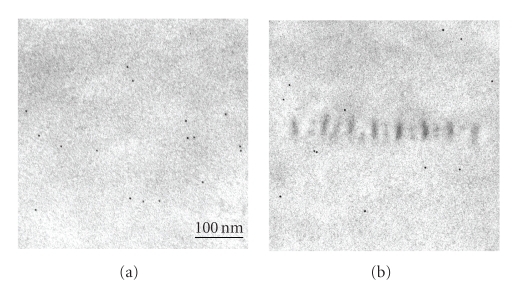
Immunoelectron-microscopic localization of laminin in Descemet's membrane by the colloidal-gold labeling method. In both the normal (a) and diabetic (b) cornea, the label was diffusely distributed in the Descemet's membrane but did not attach to the long-spacing collagen. Scale bar: 100 nm.

Next we examined the localization of the other components of the extracellular matrix, that is, laminin and type I, III, IV, and VI collagens. There is a report that type VI collagen becomes localized in the long-spacing collagen in the corneoscleral meshwork of human eyes [27]. So we examined immunoelectron-microscopically whether type VI collagen could also be detected in the long-spacing collagen of Descemet's membrane. In both the normal and diabetic cornea, the colloidal gold label was mostly seen in the corneal stroma; but none was detected in either Descemet's membrane or in the long-spacing collagen of this membrane (Figure 6). Collagens type I and III showed the same distribution as the type VI (data not shown). Laminin (Figure 7) and type IV collagen (data not shown) were localized in the amorphous material of Descemet's membrane, but were not present in the long-spacing collagen in neither the normal nor diabetic cornea.

### 3.5. Effect of antidiabetic agents on the formation of long-spacing collagen

**Figure 8:**
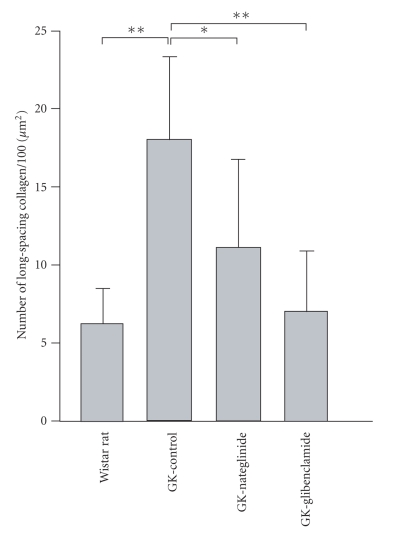
Inhibition by antidiabetic agents of the formation of long-spacing collagen in 15-week-old GK rats. The rats were administered methylcellulose alone (GK-Control), nateglinide (GK-Nateglinide) or glibenclamide (GK-Glibenclamide) for 15 weeks, which agents were suspended in methylcellulose. The number of long-spacing collagen fibrils in the Descemet's membrane was examined by electron microscopy. Data are presented as the mean ± SD (*n* = 4). GK, GK rats. *P < .05, **P < .01.

The morphological change in long-spacing collagen is thought to be one of the complications of diabetes. Thus we examined whether the abnormal formation of long-spacing collagen could be suppressed by the antidiabetic agents nateglinide and glibenclamide. As shown in our previous study [25], during the period of antidiabetic agent treatments, there was no difference in fasting blood glucose levels among vehicle-treated, nateglinide-treated, and glibenclamide-treated GK rat. Nateglinide administration reduced blood glucose levels 1 hour after feeding, whereas glibenclamide reduced blood glucose levels 2 and 3 hours after feeding, but not 1 hour after feeding [25]. Although the degree of the antidiabetic effect was different between the 2 agents, both of them significantly (*P* < .05) inhibited the formation of the long-spacing collagen in the diabetic GK rats (Figure 8). Glibenclamide treatment was more effective than nateglinide treatment (Figure 8).

## 4. DISCUSSION

Our present study revealed that a diabetes-associated increase in the number of long-spacing collagen fibrils occurred in Descemet's membrane in the cornea of type II diabetes model GK rats. This result is consistent with the findings made in the corneas of diabetic humans and type I diabetes model Streptozotocin-induced diabetic rats [11, 12]. The long-spacing collagen was much more abundant in the diabetic cornea than in the nondiabetic one, and it increased with aging in both the nondiabetic Wistar and diabetic GK rats (Figures 2 and 3). These results reveal that the age-associated morphological change in Descemet's membrane was accelerated by diabetes.

Immunoelectron microscopy showed that the long-spacing collagen contained type VIII collagen molecules (Figure 5). However, antibodies against type I, III, IV, or VI collagen did not bind to the long-spacing collagen. These results are consistent with those obtained from human and bovine Descemet's membrane [28, 29]. Type VIII collagen was found as a product of rabbit corneal and bovine aortic endothelial cells [30, 31]. Type VIII collagen is a major constituent of the hexagonal lattice of Descemet's membrane [19]. Descemet's lattice collagen can assemble into other long-spacing fibrils with a longer periodicity [19]. It is thought that the function of type VIII collagen is to provide an open, porous structure that can withstand compressive force [19]. Under some special condition of the diabetic state, type VIII collagen may contribute to the assembly of these unusual long-spacing collagen fibrils. It was earlier postulated that the aggregates of wide-spacing collagen fibrils may reflect an excessive glycosylation in diabetes [11, 12].

Long-spacing collagen is also observed in the eyes from patients with iridocorneal-endothelial syndrome, primary open-angle glaucoma, age-related macular degeneration, and Fuchs' endothelial corneal dystrophy [9, 10, 32–34]. In Fuchs' corneal dystrophy, Descemet's membrane thickens abnormally; and many long-spacing collagen fibrils are formed in its posterior layer [10, 35]. A recent study showed that Fuchs' corneal dystrophy results from a mutation in the gene encoding alpha 2 chain of type VIII collagen [36, 37]. Lack of type VIII collagen results in dysgenesis of the anterior segment of the eye, in which Descemet's membrane is markedly thinned [38]. Whereas human and mouse corneas have an anterior banded layer and a posterior unbanded layer in their Descemet's membrane, in the present study these 2 layers could not be distinguished clearly in the rat cornea. The long-spacing collagen tended to be localized in the posterior side of Descemet's membrane in the diabetic rat cornea (Figure 2). This localization is consistent with that observed in Fuch's corneal dystrophy.

Diabetes induces the dysfunction of corneal endothelium [39–42]. High glucose levels in diabetes cause the increase in sorbitol accumulation [43], advanced glycation end products [44], and O-GlcNAc-modified proteins [3] in the cornea. These changes may cause dysfunction of corneal endothelial cells and induce the morphological change of Descemet's membrane as shown in the present study. Kaji et al. [44] reported that advanced glycation end products in Descemet's membrane may be responsible for the corneal endothelial abnormalities in diabetes.

D-Phenylalanine derivative drug nateglinide and sulfonylurea drug glibenclamide are antidiabetic agents that increase insulin secretion. When we examined the effect of nateglinide and glibenclamide on the morphological changes in Descemet's membrane of GK rats, we found that the abnormal formation of the long-spacing collagen was significantly suppressed by the administration of either nateglinide or glibenclamide (Figure 8). These results suggest that control of postprandial hyperglycemia is essential to prevent the abnormal formation of long-spacing collagen in type-2 diabetes.

In the present study, glibenclamide treatment was more effective than nategelinide treatment (Figure 8). The different effects of these two antidiabetic agents may be due to their different action mechanisms. Nateglinide and glibenclamide display different effects on insulin secretion in beta cell. Our previous study showed that decreased first-phase insulin release was partially recovered when GK rats were treated with nateglinide, whereas no first-phase release occurred with glibenclamide treatment [25]. Nateglinide administration reduced blood glucose 1 hour after feeding, whereas glibenclamide administration reduced the blood glucose level 2 and 3 hours after feeding [25]. Glibenclamide treatment is more effective than nateglinide treatment in the dysfunction of second-phase insulin release. The present study suggests that glibenclamide treatment might be more effective in the inhibition of long-spacing collagen formation by recovering second-phase insulin release.

In summary, more long-spacing collagen fibrils were observed in Descemet's membrane of diabetic GK rats than in the membrane of the nondiabetic Wistar rats. Type VIII collagen was localized in the internodal region of the long-spacing collagen. Further studies are needed to elucidate the role of type VIII collagen in the formation of long-spacing collagen. Antidiabetic agents nateglinide and glibenclamide significantly suppressed the formation of the long-spacing collagen in the diabetic rats. The long-spacing collagen of the cornea would appear to be a useful indicator for studying diabetic changes in the cornea and the effect of antidiabetic agents.

## References

[1] Taylor HR, Kimsey RA (1981). Corneal epithelial basement membrane changes in diabetes. *Investigative Ophthalmology & Visual Science*.

[2] Engerman RL, Colquhoun PJ (1982). Epithelial and mesothelial basement membranes in diabetic patients and dogs. *Diabetologia*.

[3] Akimoto Y, Kawakami H, Yamamoto K, Munetomo E, Hida T, Hirano H (2003). Elevated expression of O-GlcNAc-modified proteins and O-GlcNAc transferase in corneas of diabetic Goto-Kakizaki rats. *Investigative Ophthalmology & Visual Science*.

[4] Akimoto Y, Yamamoto K, Munetomo E (2005). Elevated post-translational modification of proteins by *O*-linked *N*-acetylglucosamine in various tissues of diabetic Goto-Kakizaki rats accompanied by diabetic complications. *Acta Histochemica et Cytochemica*.

[5] Akimoto Y, Hart GW, Wells L (2007). Elevation of the post-translational modification of proteins by *O*-linked *N*-acetylglucosamine leads to deterioration of the glucose-stimulated insulin secretion in the pancreas of diabetic Goto-Kakizaki rats. *Glycobiology*.

[6] Ohguro N, Matsuda M, Ohashi Y, Fukuda M (1995). Topical aldose reductase inhibitor for correcting corneal endothelial changes in diabetic patients. *British Journal of Ophthalmology*.

[7] Meyer LA, Ubels JL, Edelhauser HF (1988). Corneal endothelial morphology in the rat. Effects of aging, diabetes, and topical aldose reductase inhibitor treatment. *Investigative Ophthalmology & Visual Science*.

[8] Schultz RO, Matsuda M, Yee RW, Edelhauser HF, Schultz KJ (1984). Corneal endothelial changes in type I and type II diabetes mellitus. *American Journal of Ophthalmology*.

[9] Levy SG, McCartney ACE, Sawada H, Dopping-Hepenstal PJC, Alexander RA, Moss J (1995). Descemet's membrane in the iridocorneal-endothelial syndrome: morphology and composition. *Experimental Eye Research*.

[10] Levy SG, Moss J, Sawada H, Dopping-Hepenstal PJC, McCartney ACE (1996). The composition of wide-spaced collagen in normal and diseased Descemet's membrane. *Current Eye Research*.

[11] Rehany U, Ishii Y, Lahav M, Rumelt S (2000). Collagen pleomorphism in Descemet's membrane of streptozotocin-induced diabetic rats: an electron microscopy study. *Cornea*.

[12] Rehany U, Ishii Y, Lahav M, Rumelt S (2000). Ultrastructural changes in corneas of diabetic patients: an electron-microscopy study. *Cornea*.

[13] Goto Y, Suzuki K, Ono T, Sasaki M, Toyota T (1988). Development of diabetes in the non-obese NIDDM rat (GK rat). *Advances in Experimental Medicine and Biology*.

[14] Bisbis S, Bailbe D, Tormo M-A (1993). Insulin resistance in the GK rat: decreased receptor number but normal kinase activity in liver. *American Journal of Physiology*.

[15] Miyamoto K, Ogura Y, Nishiwaki H (1996). Evaluation of retinal microcirculatory alterations in the Goto-Kakizaki rat. A spontaneous model of non-insulin-dependent diabetes. *Investigative Ophthalmology & Visual Science*.

[16] Sone H, Kawakami Y, Okuda Y (1997). Ocular vascular endothelial growth factor levels in diabetic rats are elevated before observable retinal proliferative changes. *Diabetologia*.

[17] Carmo A, Cunha-Vaz JG, Carvalho AP, Lopes MC (2000). Nitric oxide synthase activity in retinas from non-insulin-dependent diabetic Goto-Kakizaki rats: correlation with blood-retinal barrier permeability. *Nitric Oxide*.

[18] Wakuta M, Morishige N, Chikama T-I, Seki K, Nagano T, Nishida T (2007). Delayed wound closure and phenotypic changes in corneal epithelium of the spontaneously diabetic Goto-Kakizaki rat. *Investigative Ophthalmology & Visual Science*.

[19] Sawada H, Konomi H, Hirosawa K (1990). Characterization of the collagen in the hexagonal lattice of Descemet's membrane: its relation to type VIII collagen. *The Journal of Cell Biology*.

[20] Sawada H, Yazama F (1994). Type VI collagen in the rat testis: monoclonal antibody, isolation, and localization during development. *Biology of Reproduction*.

[21] Akimoto Y, Yamakawa N, Furukawa K, Kimata K, Kawakami H, Hirano H (2002). Changes in distribution of the long form of type XII collagen during chicken corneal development. *Journal of Histochemistry & Cytochemistry*.

[22] Akimoto Y, Kreppel LK, Hirano H, Hart GW (1999). Localization of the *O*-linked *N*-acetylglucosamine transferase in rat pancreas. *Diabetes*.

[23] Kitahara Y, Miura K, Takesue K (2002). Decreased blood glucose excursion by nateglinide ameliorated neuropathic changes in Goto-Kakizaki rats, an animal model of non-obese type 2 diabetes. *Metabolism: Clinical and Experimental*.

[24] Mine T, Miura K, Kitahara Y, Okano A, Kawamori R (2002). Nateglinide suppresses postprandial hypertriglyceridemia in Zucker fatty rats and Goto-Kakizaki rats: comparison with voglibose and glibenclamide. *Biological & Pharmaceutical Bulletin*.

[25] Kawai J, Ohara-Imaizumi M, Nakamichi Y (2008). Insulin exocytosis in Goto-Kakizaki rat *β*-cells subjected to long-term glinide or sulfonylurea treatment. *Biochemical Journal*.

[26] Ikenoue T, Okazaki K, Fujitani S (1997). Effect of a new hypoglycemic agent, A-4166 [(-)-*N*-(*trans*-4-isopropyl- cyclohexanecarbonyl)-D-phenylalanine], on postprandial blood glucose excursion: comparison with voglibose and glibenclamide. *Biological & Pharmaceutical Bulletin*.

[27] Ueda J, Yue BYJT (2003). Distribution of myocilin and extracellular matrix components in the corneoscleral meshwork of human eyes. *Investigative Ophthalmology & Visual Science*.

[28] Murata Y, Yoshioka H, Iyama K, Usuku G (1989). Distribution of type VI collagen in the bovine cornea. *Ophthalmic Research*.

[29] Marshall GE, Konstas AG, Lee WR (1991). Immunogold fine structural localization of extracellular matrix components in aged human cornea. II. Collagen types V and VI. *Graefe's Archive for Clinical and Experimental Ophthalmology*.

[30] Sage H, Trüeb B, Bornstein P (1983). Biosynthetic and structural properties of endothelial cell type VIII collagen. *The Journal of Biological Chemistry*.

[31] Benya PD, Padilla SR (1986). Isolation and characterization of type VIII collagen synthesized by cultured rabbit corneal endothelial cells. A conventional structure replaces the interrupted-helix model. *The Journal of Biological Chemistry*.

[32] Rohen JW, Lütjen-Drecoll E, Flügel C, Meyer M, Grierson I (1993). Ultrastructure of the trabecular meshwork in untreated cases of primary open-angle glaucoma (POAG). *Experimental Eye Research*.

[33] van der Schaft TL, de Bruijn WC, Mooy CM, Ketelaars DA, de Jong PT (1991). Is basal laminar deposit unique for age-related macular degeneration?. *Archives of Ophthalmology*.

[34] Roth SI, Stock EL, Jutabha R (1987). Endothelial viral inclusions in Fuchs' corneal dystrophy. *Human Pathology*.

[35] Bourne WM, Johnson DH, Campbell RJ (1982). The ultrastructure of Descemet's membrane. III. Fuch's dystrophy. *Archives of Ophthalmology*.

[36] Gottsch JD, Zhang C, Sundin OH, Bell WR, Stark WJ, Green WR (2005). Fuchs corneal dystrophy: aberrant collagen distribution in an L450W Mutant of the *COL8A2* gene. *Investigative Ophthalmology & Visual Science*.

[37] Gottsch JD, Sundin OH, Liu SH (2005). Inheritance of a novel *COL8A2* mutation defines a distinct early-onset subtype of Fuchs corneal dystrophy. *Investigative Ophthalmology & Visual Science*.

[38] Hopfer U, Fukai N, Hopfer H (2005). Targeted disruption of *Col8a1* and *Col8a2* genes in mice leads to anterior segment abnormalities in the eye. *The FASEB Journal*.

[39] Ravalico G, Tognetto D, Palomba M, Calderini S, Vattovani O (1994). Corneal endothelial function in diabetes: a fluorophotometric study. *Ophthalmologica*.

[40] Larsson L-I, Bourne WM, Pach JM, Brubaker RF (1996). Structure and function of the corneal endothelium in diabetes mellitus type I and type II. *Archives of Ophthalmology*.

[41] McNamara NA, Brand RJ, Polse KA, Bourne WM (1998). Corneal function during normal and high serum glucose levels in diabetes. *Investigative Ophthalmology & Visual Science*.

[42] Ziadi M, Moiroux P, d'Athis P, Bron A, Brun J-M, Creuzot-Garcher C (2002). Assessment of induced corneal hypoxia in diabetic patients. *Cornea*.

[43] Cisarik-Fredenburg P (2001). Discoveries in research on diabetic keratopathy. *Optometry*.

[44] Kaji Y, Amano S, Usui T (2001). Advanced glycation end products in Descemet's membrane and their effect on corneal endothelial cell. *Current Eye Research*.

